# The readiness of malaria services and uptake of intermittent preventive treatment in pregnancy in six sub-Saharan countries

**DOI:** 10.7189/jogh.14.04112

**Published:** 2024-06-28

**Authors:** Xinfang Xu, Di Liang, Jinkou Zhao, Rose Mpembeni, Joyce Olenja, Esabelle LY Yam, Jiayan Huang

**Affiliations:** 1 School of Public Health, Global Health Institute, Fudan University, Shanghai, China; 2 The Global Fund to Fight AIDS, Tuberculosis and Malaria, Geneva, Switzerland; 3 School of Public Health and Social Sciences, Muhimbili University of Health and Allied Sciences, Dar es Salaam, Tanzania; 4 Department of Public & Global Health, University of Nairobi, Nairobi, Kenya; 5 Saw Swee Hock School of Public Health, National University of Singapore, Singapore; 6 College of Health and Medicine, Australian National University, Canberra, Australia

## Abstract

**Background:**

Malaria infection during pregnancy is associated with an increased risk of maternal death, as well as adverse birth outcomes. Intermittent preventive treatment in pregnancy with sulfadoxine-pyrimethamine (IPTp-SP) is known to improve pregnancy outcomes. However, the coverage of IPTp-SP in antenatal care (ANC) in sub-Saharan Africa remains well below the target. This study aims to estimate to what extent malaria service readiness affects the uptake of IPTp-SP during ANC visits in sub-Saharan African countries.

**Methods:**

This study included 3267 pregnant women attending ANC for the first time and 2797 pregnant women who had attended ANC more than a month ago in six sub-Saharan African countries. The readiness of malaria services at each institution includes four indicators: the presence of IPTp-SP guidelines, SP availability, integration of IPTp-SP service into ANC, and provider training on IPTp-SP. The outcome variable indicates whether a pregnant woman received IPTp-SP at her current ANC visit. A modified Poisson regression model estimated the associations between malaria service readiness and IPTp-SP uptake for women eligible for the first and subsequent doses.

**Results:**

For women eligible for their first dose, visiting an institution with available SP was associated with an increased probability of receiving IPTp-SP (risk ratio (RR) = 1.43; 95% confidence interval (CI) = 1.22 to 1.67, *P* < 0.001). For women who were eligible for their next dose, the availability of SP (RR = 1.17; 95% CI = 1.04 to 1.32, *P* = 0.008) and integration of IPTp-SP service into ANC (RR = 1.82; 95% CI = 1.21 to 2.74, *P* = 0.004) in the institution were associated with increased likelihood of IPTp-SP uptake. Counterfactual predictions indicated that enhanced provider training could boost IPTp-SP uptake in high-uptake countries, while better SP availability and IPTp-SP integration into ANC would significantly impact low-uptake countries.

**Conclusions:**

For better IPTp-SP coverage, strategies should be customised. High uptake countries should focus on provider training, while low uptake ones should ensure IPTp-SP availability and service integration.

With 608 000 deaths annually in 2022, malaria remains a major public health challenge in sub-Saharan Africa over the past decade, despite worldwide efforts to eliminate it [1]. The burden of malaria has intensified, particularly in the aftermath of the COVID-19 pandemic and the expansion of mosquito habitats due to climate changes [2,3]. Malaria is mostly found in tropical countries and poses the greatest risk to vulnerable populations, such as pregnant women, young children, international tourists, and people living with HIV [1].

While malaria infection is a lifelong threat, infection during pregnancy poses substantial risks not only to the mother but also to the offspring [4,5]. A study found that pregnant women are more susceptible to malaria than non-pregnant women because they are less immune and more attractive to mosquitoes during pregnancy [6]. Malaria during pregnancy peaks between the 13th and 16th gestational weeks [7] and is associated with an increased risk of anemia, maternal death, and adverse birth outcomes such as miscarriage and low birth weight [8,9]. Therefore, malaria prevention is very important during this period.

To mitigate the serious health consequences of malaria infection during pregnancy, it is recommended that malaria prevention be integrated into antenatal care (ANC) in malaria-endemic areas, particularly sub-Saharan Africa [10]. Since 2004, the World Health Organization (WHO) has recommended that women receive a minimum of two doses of intermittent preventive treatment in pregnancy (IPTp) [11]. In 2012, the policy was updated to recommend that sulfadoxine-pyrimethamine (SP), an anti-malarial drug considered safe and effective for pregnant women, should be administered at every ANC visit starting as early as possible in the second trimester, provided that doses are at least one month apart [12,13]. In 2022, the policy was further updated to recommend the delivery of IPTp-SP to all pregnant women, regardless of the number of pregnancies [1]. Regardless of whether a hospital has a stock of SP, health providers performing prenatal care should prescribe the drug to pregnant women who are eligible for IPTp.

Although 35 countries in sub-Saharan Africa have integrated malaria prevention into ANC as a matter of policy, a gap remains between IPTp-SP service and ANC for pregnant women in the region due to inadequate health care infrastructure, limited resources, cultural practices, and socioeconomic factors. While 78% of pregnant women in sub-Saharan Africa used ANC services at least once in 2022, only 64% received at least one dose of IPTp-SP. Furthermore, only 42% of pregnant women met the WHO recommendation to receive at least three doses of IPTp-SP [14].

Previous studies have found that the uptake of IPTp-SP is affected by factors from both the demand and supply sides. Many demand-side barriers, such as a woman’s level of education [15], number of pregnancies [16], and trust in the health care system [17], are difficult to modify in the short term. This is especially the case in sub-Saharan Africa, due to cultural and resource constraints. These barriers also hinder the uptake of IPTp in sub-Saharan Africa from improving demand-side factors. As IPTp-SP is usually prescribed and administered under direct observation of health care providers, supply-side barriers, such as stockouts or lack of experienced health care providers, were also critical for effective interventions. Studies examining institutional-level factors have drawn attention to measures of malaria service readiness, as the mere availability of malaria services does not guarantee readiness in the provision of IPTp [18,19]. According to WHO, malaria service readiness refers to the capacity of health institutions to provide malaria services, including the presence of trained staff, malaria-related guidelines, equipment, diagnostic capacity, and medicines [20].

There are still gaps in understanding barriers to increasing IPTp uptake rates. Previous studies on the relationship between malaria service readiness and IPTp uptake are limited to specific countries, making it uncertain whether findings are applicable in different settings. Second, there is limited information regarding whether service readiness factors affect the initiation and continuation of IPTp-SP uptake. A recent study suggested that women might have different trajectories in receiving IPTp doses, and some might be more likely to stay on track in receiving optimal doses of IPTp [21]. However, it is unknown whether malaria service readiness could influence the trajectories. This study investigates the link between IPTp-SP readiness in health care institutions and its uptake among eligible women in six sub-Saharan countries. It hypothesises that improved malaria service readiness correlates with higher IPTp uptake during ANC visits.

## METHODS

### Study Design and Data Source

This cross-sectional study was based on the data from the Service Provision Assessment (SPA) in six African countries: Kenya (2010), Senegal (2014, 2016, 2018), Democratic Republic of Congo (DRC) (2017 - 2018), Malawi (2013 - 2014), Namibia (2009) and Tanzania (2014 - 2015). We selected the most recent data available for these countries. Based on data availability, we selected all sub-Saharan African countries with SPA data, prioritising the most recent data while minimising the difference in data years. To increase the sample size, we incorporated data from closely spaced years in Senegal into our study. Data are available at https://www.dhsprogram.com/data/available-datasets.cfm.

SPA is a standardised survey assessing service readiness in health systems, conducted on a nationally representative sample of health care institutions using a master list. Each SPA survey consists of four phases. The first stage is survey preparation and questionnaire design. The SPA questionnaires include four parts: the SPA institution questionnaire, provider questionnaire, antenatal client exiting interview questionnaire, and ANC visit protocol observation. All questionnaires are designed following the harmonised standards of the SPA and are adjusted to suit the implementation in each country. The second phase involves training and fieldwork. All staff participating in the survey are uniformly trained before the fieldwork, to ensure that the results are consistent and reliable in each country and each institution. In surveyed health institutions, the institution questionnaire was completed by a manager or designated knowledgeable person, while the provider questionnaire was filled by a sample of health care providers present during the assessment day and involved in surveyed services. Additionally, ANC clients were systematically selected for client exit interviews as well as observation, based on the number of clients present at each service site on the day of the visit, with a maximum of five clients per provider and up to 15 observations per service in an institution. The last two stages are data processing and reporting, including data entry, coding, verification, and final report formation, which are completed by professional staff by the unified standards of SPA [22].

The data we used in this study are publicly available and properly anonymised. Informed consent was obtained at the time of original data collection by DHS. The analysis did not involve any secondary use of data. No individuals or biological samples were contacted for this study. Thus, ethical approval did not apply to this study.

### Study sample

The study population of this study comprised pregnant women who were eligible for their first or subsequent dose of IPTp-SP during their ANC visit. According to the WHO Recommendations, pregnant women in malaria-endemic areas should start taking IPTp-SP every other month from the beginning of the second trimester (13 weeks of gestation) to prevent malaria infection during pregnancy. Therefore, we first identified 15 553 pregnant women and then limited the sample to 10 535 women whose gestational age was 13 weeks or more. They were further categorised into two groups to ensure that the surveyed clients were eligible to receive IPTp-SP at the time of the survey. The first group comprised 4530 women who were visiting ANC for the first time and had no history of IPTp-SP uptake, to ensure that they were required to receive their first dose of IPTp during this ANC visit. The second group consisted of 3334 women who had attended ANC more than a month ago and should also receive a subsequent dose of IPTp during this ANC visit. After removing all the sample data with missing values, the final sample size for the first group was 3267 pregnant women, and for the second group, it was 2797. The sample inclusion process is illustrated in Figure S1 in the **Online Supplementary Document**.

### Definition of variables and covariates

Based on Anderson’s behavior model, this study selects variables from three aspects: predisposing, enabling, and need factors [23]. According to the model, predisposing factors include social and demographic structures, while enabling factors facilitate service utilisation through resource accessibility (income and availability of services). Need factors, such as disease or physical conditions, drive individuals to seek services.

#### Outcome variable

The outcome variable indicates whether a pregnant woman received any IPTp-SP at their current ANC visit. It is recommended that health providers should prescribe drugs whether they are available in institutions or not. Therefore, this variable was determined by two questions in the questionnaire: ‘whether the provider gave malaria prophylaxis medicine (SP) to the client during the consultation’ and ‘whether the provider prescribed malaria prophylaxis medicine (SP) to the client to obtain elsewhere’. The variable was assigned a value of 1 if the answer to either of these questions was ‘yes’. Otherwise, it was assigned a value of 0.

#### The primary predictor of interest

The primary predictor of interest is the malaria service readiness at each institution. Based on the WHO definition and data accessibility, the malaria service readiness in ANC settings was measured from the following four dimensions: integration of IPTp-SP service into ANC, availability of SP, the presence of IPTp-SP guidelines, and provider training on IPTp-SP.

The integration of IPTp-SP service into ANC was measured by a binary variable indicating whether ANC providers provide IPTp-SP service to pregnant women as part of routine ANC. This indicator is used to assess whether the organisation is following the WHO's recommendations in formulating policies.

The availability of SP was measured by a binary variable indicating whether SP was observed in the institution on the day of the survey. This indicator is an assessment of whether an institution has a stock of medicines.

The presence of IPTp-SP guidelines was measured by a binary variable indicating whether the institution had national guidelines for malaria diagnosis, treatment, and prevention.

Provider training on IPTp-SP was used to assess the level of malaria-related training received by health service providers within an institution. First, the proportion of providers who have received training in institutions was calculated based on a question about IPTp-related training in the questionnaire. Institutions were then ranked and divided into four quantiles based on these percentages: the lowest quantile (0–25%), the second quantile (25–50%), the third quantile (50–75%), and the highest quantile (75–100%).

#### Covariates

To control for confounding, the regression models included variables at the individual levels, institutional levels, a binary variable indicating the rainy season, and country-fixed effects.

At the individual level, variables included age, birth history, education, doses of IPTp-SP received during pregnancy, gestational age, intention to give birth at the health institution where ANC was received on the day of the survey, and the type of health service provider.

Birth history was measured by a binary variable indicating whether the current one was the first pregnancy. Education and intention to give birth at the health institution where ANC was received were measured by binary variables indicating whether the woman had ever attended school and whether women planned to give birth at the ANC institutions, respectively. Gestational age was measured by a categorical variable with seven categories (13–16 weeks, 17–20 weeks, 21–24 weeks, 25–28 weeks, 29–32 weeks, 33–36 weeks, >37 weeks). The type of health service provider was measured by a categorical variable indicating whether the provider at the ANC visit was a doctor, nurse, or other type of provider. Other variables were treated as continuous variables.

At the institutional level, variables included institution type, managing authority, and number of hospital beds for maternity and delivery in the institution. The type of institution was measured by a categorical variable with four categories (hospital, clinic, health center, dispensary). The ownership of institutions was measured by a three-category variable (government/public, private, others). The number of hospital beds for maternity and delivery was treated as a continuous variable that describes the number of institutional beds.

### Statistical analysis

To directly estimate RRs, we used a modified Poisson regression model to analyse the association of the readiness of institutional malaria services with women's receipt of IPTp-SP in their ANC visits. Negative Binomial Regression results showed that the null hypothesis of overdispersion (α = 0) was rejected (*P* > 0.05), so the choice of the Poisson model is reasonable. In this count model, a Poisson model with robust standard errors can obtain valid statistical inference when fitting binary data [24–26]. We also performed stratified analyses by country.

In addition, we calculated the marginal effects of the statistically significant variables in the regression model. Marginal effects are counterfactual predictions that can help interpret models in the scale of interest. In this study, marginal effects were used to predict the percentage change in IPTp-SP uptake rates if a measure of malaria service readiness was set to specific values.

Nationally representative weighted data were used to adjust for sampling weight and clustering effects. A *P*-value <0.05 was considered statistically significant. Stata version 17.0 was used to conduct the data analysis.

## RESULTS

### Characteristics of respondents

The study sample comprised two groups of women: 3267 women who had not received ANC and IPTp-SP before and 2797 women who had received their last ANC more than one month before. In the first group, the average age was 25.56 years. Among them, 73.95% had been pregnant before, and 81.98% expressed their intention to give birth in the same health institution where they received ANC. More than 85% of these women were between 13 and 28 weeks of gestation. The second group had similar individual characteristics to the first group, except that they were more likely to be beyond 28 weeks of pregnancy (**Table 1**).

**Table 1:** Characteristics of two sample groups at the individual level

	Women visiting ANC for the first time without IPTp history (n = 3267)		Women who had been present for ANC for more than one month before (n = 2797)	
	**Mean/n**	**SD/%**	**Mean/n**	**SD/%**
Age, in years	25.56	6.39	25.75	6.29
Provider category				
*Doctor*	110	3.37	33	1.18
*Nurse*	3040	93.05	2705	96.71
*Other*	117	3.58	59	2.10
Birth history				
*Not first-time pregnancy*	2416	73.95	2047	73.19
*First-time pregnancy*	851	26.05	750	26.81
Education level				
*Never attended school*	612	18.73	669	23.92
*Ever attended school*	2655	81.27	2128	76.08
Intention to give birth at the institution where receiving ANC				
*Will not delivery in the same institution*	588	18.02	559	19.99
*Will delivery in the same institution*	2679	81.98	2238	80.01
Gestational age, in years				
*13–16*	451	13.8	49	1.75
*17–20*	791	24.21	148	5.29
*21–24*	860	26.32	348	12.44
*25–28*	707	21.64	607	21.70
*29–32*	315	9.64	737	26.35
*33–36*	108	3.31	649	23.20
*>37*	35	1.07	259	9.26
Season				
*Wet season*	1962	60.06	1856	66.36
*Dry season*	1305	39.94	941	33.64
Number of SP doses received during pregnancy *	/	/	1.47	1.01

ANC – antenatal care, IPTp – intermittent preventive treatment in pregnancy, SD – standard deviation, SP – sulfadoxine-pyrimethamine

*The first sample was pregnant women who attended ANC for the first time and had no history of SP, so the indicator ‘SP dose received during pregnancy’ was only included in the model of the second sample.

The total number of institutions included in the study was 1923, of which 30.73% were hospitals and 46.80% were health centres. Concerning the ownership of institutions, 75.98% were public institutions. Regarding the readiness of malaria services in health institutions, SP was not available in 29.02% of the institutions, and IPTp was not incorporated into the ANC routine in 9.57% of the institutions (**Table 2**).

**Table 2:** Characteristics of institutions (n = 1923) included in the study

	Mean/n	SD/%
Institution type		
*Hospital*	591	30.73
*Clinic*	217	11.28
*Health center*	900	46.80
*Dispensary*	215	11.18
Managing authority (ownership)		
*Government/public*	1461	75.98
*Private*	207	10.76
*Others*	255	13.26
Number of maternity beds	11.77	18.87
Number of delivery beds	2.32	2.10
Availability of SP		
*Not available*	558	29.02
*At least one valid*	1365	70.98
Integration of IPTp-SP service into ANC		
*No*	184	9.57
*Yes*	1739	90.43
Presence of IPTp-SP guidelines		
*No*	822	42.75
*Yes*	1101	57.25
Four quartiles of provider training coverage on IPTp-SP		
*Lowest quartile*	1389	72.23
*Second quartile*	418	21.74%
*Third quartile*	72	3.74
*Highest quartile*	44	2.29

ANC – antenatal care, IPTp – intermittent preventive treatment in pregnancy, SD – standard deviation, SP – sulfadoxine-pyrimethamine

### Uptake of IPTp-SP in six countries

**Figure 1:**
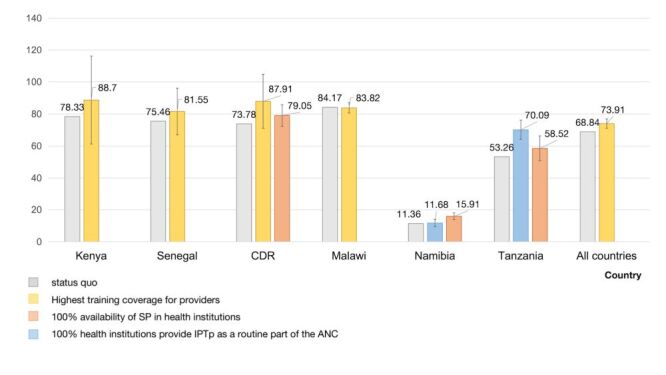
Changes in IPTp uptake rates among pregnant women visiting ANC for the first time due to factors of readiness of malaria-related services in different countries. Models adjusted for age, birth history, education, doses of IPT-SP received during pregnancy, gestational age, intention to give birth at the health institution where ANC was received on the day of the survey, the type of health service provider, facility type, managing authority, number of hospital beds for maternal and delivery in the institutions. Shown in the figures are statistically significant variables in each country model. ANC – antenatal care, IPTp – intermittent preventive treatment in pregnancy, SP – sulfadoxine-pyrimethamine

**Figure 2:**
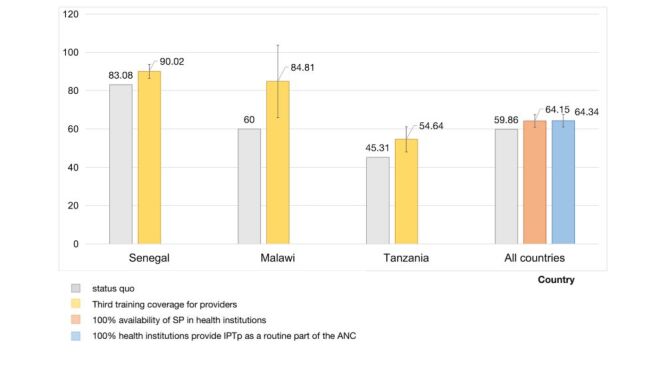
Changes in IPTp uptake rates among pregnant women being present for ANC for more than one month due to factors of readiness of malaria related services in different countries. Models adjusted for age, birth history, education, doses of IPT-SP received during pregnancy, gestational age, intention to give birth at the health institution where ANC was received on the day of the survey, the type of health service provider, facility type, managing authority, number of hospital beds for maternal and delivery in the institutions. Shown in the figures are statistically significant variables in each country model. ANC – antenatal care, IPTp – intermittent preventive treatment in pregnancy, SP – sulfadoxine-pyrimethamine

The current uptake of IPTp-SP among pregnant women in six countries during the study year is shown in **Figure 1** and **Figure 2**. The study revealed significant variations in IPTp-SP uptake rates among pregnant women across these countries. The uptake of IPTp-SP ranges from 70 to 85% for pregnant women attending ANC for the first time in Kenya, Senegal, Malawi, and DRC. Notably, in Namibia and Tanzania, only 11.36 and 53.25% of women received SP during pregnancy in their first ANC visit, respectively. For pregnant women who had visited ANC more than one month apart, 83% in Senegal, 60% in Malawi, and only 45.31% in Tanzania received SP during their ANC visit.

### Institutional factors influencing the uptake of IPTp-SP

The associations between institutional factors and the uptake of IPTp-SP for the two groups of women are presented in **Table 3**. The complete regression results can be found in Table S1 in the **Online Supplementary Document**.

**Table 3:** Institutional factors influencing IPTp-SP in two group*

	Women receiving ANC for the first time			Women who attended ANC for more than one month		
	**Regression results**		**Marginal effects**	**Regression results**		**Marginal effects**
	**RR (95% CI)**	***P*-value**	**Predicted probability (95% CI)**	**RR (95% CI)**	***P*-value**	**Predicted probability (95% CI)**
**Presence of IPTp-SP guidelines**						
No	(Reference)		(Reference)	(Reference)		(Reference)
Yes	1.05 (0.96, 1.15)	0.270	0.03 (−0.03, 0.09)	1.02 (0.92, 1.13)	0.682	0.01 (−0.05, 0.07)
**Availability of SP**						
Not available	(Reference)		(Reference)	(Reference)		(Reference)
At least one valid	1.43 (1.22, 1.67)	<0.001	0.22 (0.13, 0.31)	1.17 (1.04, 1.32)	0.008	0.12 (0.06, 0.18)
**Integration of IPTp-SP service into ANC**						
No	(Reference)		(Reference)	(Reference)		(Reference)
Yes	1.35 (0.99, 1.82)	0.051	0.18 (0.02, 0.34)	1.82 (1.21, 2.74)	0.004	0.31 (0.18, 0.45)
**Four quantiles of provider training coverage on IPTp-SP**						
Lowest quartile	(Reference)		(Reference)	(Reference)		(Reference)
Second quartile	1.08 (0.98, 1.19)	0.115	0.05 (−0.01, 0.12)	1.02 (0.93, 1.12)	0.653	0.02 (−0.04, 0.08)
Third quartile	1.11 (0.91, 1.37)	0.259	0.08 (−0.07, 0.23)	1.04 (0.88, 1.22)	0.652	0.04 (−0.07, 0.15)
Highest quartile	1.01 (0.83, 1.22)	0.978	0.00 (−0.17, 0.13)	0.83 (0.67, 1.03)	0.098	−0.07 (−0.18, 0.05)

ANC – antenatal care, CI – confidence interval, IPTp – intermittent preventive treatment in pregnancy, RR – risk rato, SP – sulfadoxine-pyrimethamine

*Models adjusted for age, birth history, education, doses of IPTp-SP received during pregnancy, gestational age, intention to give birth at the health institution where ANC was received on the day of the survey, the type of health service provider, institution type, managing authority, number of hospital beds for maternal and delivery in the institutions.

In the first group, pregnant women who received ANC in institutions where SP was available on the day of the survey were more likely to take IPTp-SP than those who received ANC in institutions where SP was not available (risk ratio (RR) = 1.43; 95% confidence interval (CI) = 1.22 to 1.67, *P* < 0.001). The average marginal effect of SP availability in institutions was 0.22 (95% CI = 0.13 to 0.31), indicating a 22 percentage points increase in IPTp-SP uptake among women when all health institutions guaranteed access to SP medicines, compared to the scenario where SP was not available in all health institutions (**Table 3**).

In the second group, pregnant women were more likely to receive IPTp-SP if SP was available in the institutions on the day of the survey (RR = 1.17; 95% CI = 1.04 to 1.32, *P* = 0.008) or if the institutions provided IPTp-SP as part of routine ANC (RR = 1.82; 95% CI = 1.21 to 2.74, *P* = 0.004). The average marginal effect of SP availability in institutions was 0.12 (95% CI = 0.06 to 0.18), which means a 12 percentage points increase in IPTp-SP uptake among women when all health institutions guaranteed access to SP medicines. It's also worth noting that the average marginal effect of providing IPTp-SP as part of routine ANC was 0.31 (95% CI = 0.18 to 0.45). In other words, if all health institutions use IPTp-SP as ANC routine, the percentage of pregnant women receiving IPTp-SP at ANC is 31 percentage points higher than if all health institutions do not use IPTp-SP as ANC routine (**Table 3**).

### Institutional factors influencing the uptake of IPTp-SP across countries

The counterfactual uptake rates of SP among pregnant women across countries is shown in **Figure 1** and **Figure 2**, in response to altered levels of institutional readiness. Results found that regions with high IPTp-SP uptake show greater influence from provider training. In contrast, in areas with low uptake, SP storage and integration of IPTp-SP service into ANC play bigger roles.

In the first group, the percentage of pregnant women receiving IPTp-SP would increase from 78.33 to 88.7% in Kenya (increased by 13.24% from the current level), from 73.78 to 87.91% in DRC (increased by 19.15% from the current level), and from 75.46 to 81.65% in Senegal (increased by 8.20% from the current level), when the coverage of provider training in all institutions was increased to more than 75%. For Tanzania, the percentage of pregnant women using SP would increase from 53.26 to 58.52% and 70.09%, respectively, if all institutions had adequate SP stocks (increased by 9.88% from the current level) and if all institutions used IPTp as ANC routine (increased by 31.64% from the current level) (**Figure 1**).

This trend was also consistent in the second group. The percentage of pregnant women receiving IPTp-SP will increase from 60.00 to 84.81% (increased by 41.35% from the current level) in Malawi when the coverage of provider training of IPTp-SP in all institutions is increased to 50–75%. In Tanzania, the percentage of pregnant women using SP would increase from 45.31 to 54.64% if all institutions had adequate SP stocks (increased by 20.59% from the current level) (**Figure 2**).

## DISCUSSION

Discuss your results here and address their importance, as well as limitations. You can use the subheadings as in the Methods and Results sections While IPTp-SP uptake among pregnant women in sub-Saharan Africa has increased, a notable gap still exists between ANC participation rates and actual IPTp-SP uptake [27]. Significant variations in IPTp-SP uptake rates among pregnant women were also observed across countries. According to our results, the readiness of malaria services is positively associated with the uptake of IPTp-SP among pregnant women in ANC who should receive IPTp-SP. While older data from some countries limits our study, our findings still have implications for increasing IPTp uptake in sub-Saharan African countries. The research findings from Kenya's 2018-2019 KHFA report and the 2023 Malaria Report highlight the continued low IPTp acceptance rates and the deficient level of malaria service readiness in sub-Saharan African countries [28]. These observations remain in alignment with our study's findings. Consequently, the strategies proposed in our study to increase IPTp uptake by ameliorating malaria service readiness are still pertinent and applicable in the current context.

Consistent with our hypothesis, the readiness of malaria services is positively associated with a positive effect on the uptake of IPTp-SP among pregnant women in ANC who should receive IPTp-SP. The results align with preexisting studies [29–31] that highlight the importance of health institution preparedness and service accessibility in ensuring IPTp-SP uptake in pregnant women. Our study is unique in focusing specifically on pregnant women eligible for IPTp-SP, analysing the separate factors influencing both the start and continuation of IPTp-SP usage. According to the results, the uptake rates of IPTp in Tanzania and Namibia were significantly lower than those in the other four countries. This may be because these two countries use a different IPTp schedule than the one recommended by the WHO (administering IPTp between 20–24 and 28–32 weeks of gestation for Tanzania [32] and between 26–28 and 34–36 weeks of gestation for Namibia [33]). In contrast, Malawi and Kenya were the first countries to implement the WHO IPTp policy [34]. A systematic review of sub-Saharan African countries found that, in addition to lagging policies, Tanzania's health system suffers from a lack of leadership and inadequate funding. Issues such as slow decentralisation processes for programme management and the need to pay ANC fees have kept IPTp-SP uptake low in the country [35].

Our cross-country predictions showed that enhancing provider training coverage to the highest quartile level could significantly boost IPTp-SP uptake in countries with already high usage. Conversely, increasing SP availability and integrating IPTp-SP into ANC services would be more effective in countries with low initial uptake.

### Positive association of the stocks of SP with the uptake of IPTp-SP

SP availability at health institutions significantly influences both the initiation and continuation of IPTp-SP uptake among pregnant women, aligning with previous research findings [36]. However, consistently providing SP remains a challenge in some countries [37]. In our study, 26.91% of health institutions lacked SP during women's first ANC visits. When ANC institutions are unable to provide SP, pregnant women often resort to suboptimal preventive measures or no preventive measures at all. Pregnant women who do not receive IPTp-SP at the recommended intervals increase their risk of malaria infection.

Country-specific analysis revealed that in nations with low IPTp-SP uptake, ensuring sufficient SP drug storage is a key strategic focus. For instance, in Tanzania, if all facilities had adequate stocks of SP drugs, the percentage of pregnant women who received IPTp-SP again after the first dose would increase by more than 20 percent from current levels. This may be because SP is still insufficient in these countries, which reflects a weakness in the health system [38]. Therefore, special attention should be given to addressing the financial and supply chain challenges that hinder the timely procurement of SP drugs, as seen in Tanzania, to ensure a steady supply and thereby elevate IPTp-SP uptake rates among pregnant women.

### Integration of IPTp-SP service into ANC can also increase the uptake of IPTp-SP in non-first ANC visitors’ groups

In addition, the regular provision of IPTp-SP as part of ANC is positively associated with the initiation of IPTp-SP uptake, which has also been suggested by some previous studies [39]. Counterfactual predictions suggested that if all institutions in the six countries integrated IPTp-SP into ANC, the uptake of IPTp-SP among pregnant women without IPTp-SP history would increase. For instance, in Tanzania, if all facilities integrated IPTp-SP into ANC, the percentage of pregnant women who were the first for their ANC visits would increase by more than 30 percent from current levels. Health service providers in health institutions that do not include IPTp-SP as a routine part of ANC often do not proactively mention IPTp-SP to pregnant women, thereby reducing the probability of pregnant women receiving perinatal malaria prevention information and care. Hence, in nations with low uptake rates, such as Tanzania and Namibia, a two-pronged strategy should be employed: increasing the storage of SP drugs and integrating IPTp-SP into the ANC routine.

### Boosting training enhances IPTp-SP uptake for pregnant women in countries with high SP uptake rates

In the main regression models, the association between health provider training coverage and outcomes was not significant. However, the country-specific analysis showed that enhancing provider training boosts IPTp uptake in high uptake countries. Training optimises implementation, keeping health care professionals updated on malaria prevention in pregnancy.

To enhance IPTp-SP uptake rates in sub-Saharan countries, it is imperative to adopt tailored strategies for different nations. In countries with a high prevalence of IPTp-SP uptake, such as Kenya, Senegal, and Malawi, the focus should be on further optimising the implementation process through advanced training of health providers on IPTp-SP protocols. At the same time, our findings also suggest that strengthening health systems and services in sub-Saharan Africa remains a top priority. In the future, more funding and support should be used to strengthen the integrity of health systems and access to health services.

### Strengths

Our study examined eligible pregnant women for IPTp-SP during ANC visits in six countries, analysing both personal and institutional factors. It considered factors influencing both the start and continuation of IPTp. We used nationally representative weighted data to ensure results reflect each country's situation and provide a basis for comparison within the sub-Saharan region.

### Limitations

Our analysis was based on retrospective cross-sectional survey data; therefore, we cannot establish causation and avoid uncontrolled confounding factors. However, the SPA data used in this study came from the health service provider side, so most of the data were based on actual observations rather than self-reported information which are prone to recall bias and social desirability bias. For example, the outcome variable and the other three indicators of malaria service readiness except Provider training on IPTP-SP are all actual observation variables. Second, we assumed women prescribed SP would comply, which may affect our results if compliance is low. Thus, our findings represent a best-case probability scenario [40].

The data we used spans from 2009 to 2018, during which period the eligibility criteria, starting point, and time interval of administering IPTp remain unchanged. The only policy change occurred in 2012, when the World Health Organization (WHO) recommended that pregnant women receive at least three doses of IPTp, as opposed to the previously recommended two. In this study, all SPA data were collected after the respective countries adopted the WHO 2012 guidance, except for Kenya (2010) and Namibia (2009) [41]. This policy change should not impact our first analysis sample (those visiting ANC for the first time with no history of IPTp-SP) as the eligibility criteria remained the same during the study period. Furthermore, while the policy change may affect our second analysis sample (those who attended ANC more than a month ago and should have also received IPTp during these visits), Kenya (2010) and Namibia (2009) were excluded from this analysis due to data availability issues. Therefore, all countries included in the second sample analysis had previously adopted the WHO 2012 guidance. However, the time span may cause other influencing factors that we cannot control, which is also one of our limitations.

In WHO Recommendation 2015 and previous editions, it was recommended that health facilities should provide IPTp to all women in their first and second pregnancies, but in the updated 2022 guidelines, IPTp-SP is now recommended for all pregnant women, regardless of the number of pregnancies. However, due to the lack of data related to pregnancy parity in the SPA database, we were unable to include parity in our study and combine the different versions of the guidelines for discussion.

## CONCLUSIONS

Increased IPTp-SP uptake among pregnant women is linked to SP availability and its integration into ANC routines. However, institutional readiness factors vary by country, suggesting the need for country-specific strategies.

## Additional material


Online Supplementary Document
