# Supplementary material for: The readiness of malaria services and uptake of intermittent preventive treatment in pregnancy in six sub-Saharan countries
Source: J Glob Health. 2024 Jun 28;14:04112. doi: 10.7189/jogh.14.04112 (PMC11211972; doi:10.7189/jogh.14.04112)
Supplement: Online Supplementary Document [file jogh-14-04112-s001.pdf]

ONLINE SUPPLEMENTARY DOCUMENT

Title: The Readiness of Malaria Services and Uptake of Intermittent Preventive Treatment in Pregnancy in Six sub-Saharan Countries

Xinfang Xu, MPH[1]*;

Di Liang, Ph.D.[1]*;

Jinkou Zhao, Ph.D.[2];

Rose Mpembeni, Ph.D.[3];

Joyce Olenja, Ph.D.[4];

Esabelle LY Yam, Ph.D.[5,6];

Jiayan Huang, Ph.D.[1]†

* Joint first authorship.

† Correspondence: jiayanhuang@fudan.edu.cn

[1] Shanghai Institute of Infectious Disease and Biosecurity, School of Public Health, Fudan University, Shanghai 200032, China
[2] The Global Fund to Fight AIDS, Tuberculosis and Malaria, Geneva, Switzerland
[3] School of Public Health and Social Sciences, Muhimbili University of Health and Allied Sciences, Dar es Salaam, Tanzania
[4] Department of Public & Global Health, University of Nairobi, Nairobi, Kenya
[5] Saw Swee Hock School of Public Health, National University of Singapore, Singapore
[6] College of Health and Medicine, Australian National University, Canberra, Australia

Table S1. Factors influencing IPT in women receiving ANC for two sampling groups.

	Women visiting ANC for the first time without IPT history		Women who had been present for ANC for more than one month	
	RR (95% CI)	P value	RR (95% CI)	P value
institution level factors				
**institution type**				
Hospital	[Reference]		[Reference]	
Clinic	0.95 (0.78~1.14)	0.562	0.96 (0.82~1.13)	0.632
Health center	0.90 (0.83~0.99)	0.025	0.99 (0.86~1.14)	0.906
Dispensary	0.90 (0.76~1.06)	0.214	0.87 (0.69~1.10)	0.251
**Managing authority (ownership)**				
Government/public	[Reference]		[Reference]	
Private	0.86 (0.77~0.97)	0.017	1.14 (0.97~1.32)	0.103
Other	0.87 (0.73~1.02)	0.084	0.90 (0.67~1.22)	0.503
**Numbers of maternity bed**	0.99 (0.99~1.00)	0.500	1.00 (0.99~1.00)	0.955
**Numbers of delivery bed**	0.98 (0.96~1.00)	0.015	1.00 (0.97~1.03)	0.911
**Have IPTp-SP guidelines in the institution**				
No	[Reference]		[Reference]	
Yes	1.05 (0.96~1.15)	0.270	1.02 (0.92~1.13)	0.682
**SP available in the institution**				
Not available	[Reference]		[Reference]	
At least 1 valid	1.43 (1.22~1.67)	<0.001	1.17 (1.04~1.32)	0.008
**Provide IPT as part of routine ANC**				
No	[Reference]		[Reference]	
Yes	1.35 (0.99~1.82)	0.051	1.82 (1.21~2.74)	0.004
**4 quartiles of IPT training share**				
Lowest quartile	[Reference]		[Reference]	
Second quartile	1.08 (0.98~1.19)	0.115	1.02 (0.93~1.12)	0.653
Third quartile	1.11 (0.91~1.37)	0.259	1.04 (0.88~1.22)	0.652
Highest quartile	1.01 (0.83~1.22)	0.978	0.83 (0.67~1.03)	0.098
Individual level factors				
**Age**	1.00 (0.99~1.01)	0.416	1.00 (0.99~1.00)	0.411
**Provider category**				
Doctor	[Reference]		[Reference]	
Nurse	1.19 (0.87~1.62)	0.278	1.90 (1.03~3.51)	0.040
Other	1.17 (0.83~1.66)	0.367	1.80 (0.89~3.63)	0.103
**Birth history**				
Not first time pregnancy	[Reference]		[Reference]	

First time pregnancy	0.97	(0.89~1.05)	0.422	0.97	(0.88~1.07)	0.553
**Education**						
Never attended school		[Reference]			[Reference]	
Ever attended school	1.01	(0.92～1.10)	0.894	0.95	(0.87~1.03)	0.174
**Intention to give birth at the institution where receiving ANC**						
Not delivery here		[Reference]			[Reference]	
Will delivery here	1.03	(0.92~1.14)	0.618	0.94	(0.84~1.06)	0.553
**Gestational age**						
13-16		[Reference]			[Reference]	
17-20	1.51	(1.31~1.75)	<0.001	1.18	(0.92~1.52)	0.190
21-24	1.74	(1.51~2.00)	<0.001	1.18	(0.93~1.48)	0.173
25-28	1.77	(1.53~2.05)	<0.001	1.18	(0.93~1.48)	0.168
29-32	1.87	(1.59~2.19)	<0.001	0.99	(0.78~1.25)	0.915
33-36	1.26	(0.94~1.68)	0.118	0.79	(0.61~1.01)	0.055
>37	1.38	(0.77~2.48)	0.275	0.54	(0.41~0.72)	<0.001
**Season**						
Wet season		[Reference]			[Reference]	
Dry season	1.03	(0.95~1.13)	0.458	0.94	(0.88~1.01)	0.105
***Number of SP doses received during pregnancy**		/	/	1.22	(1.16~1.28)	<0.001
**Country**						
Kenya		[Reference]			/	
Senegal	1.19	(0.97~1.46)	0.100		[Reference]	
CDR	1.15	(0.96~1.38)	0.118		/	
Malawi	1.28	(1.09~1.51)	0.003	0.63	(0.56~0.70)	<0.001
Namibia	0.20	(0.09~0.41)	0.000		/	
Tanzania	1.05	(0.85~1.29)	0.658	0.68	(0.59~0.78)	<0.001

Abbreviation: ANC, antenatal care; IPTp, intermittent preventive treatment in pregnancy; SP, sulfadoxine-pyrimethamine.

*The first sample was pregnant women who attended ANC for the first time and had no history of SP, so the indicator "SP dose received during pregnancy" was only included in the model of the second sample.

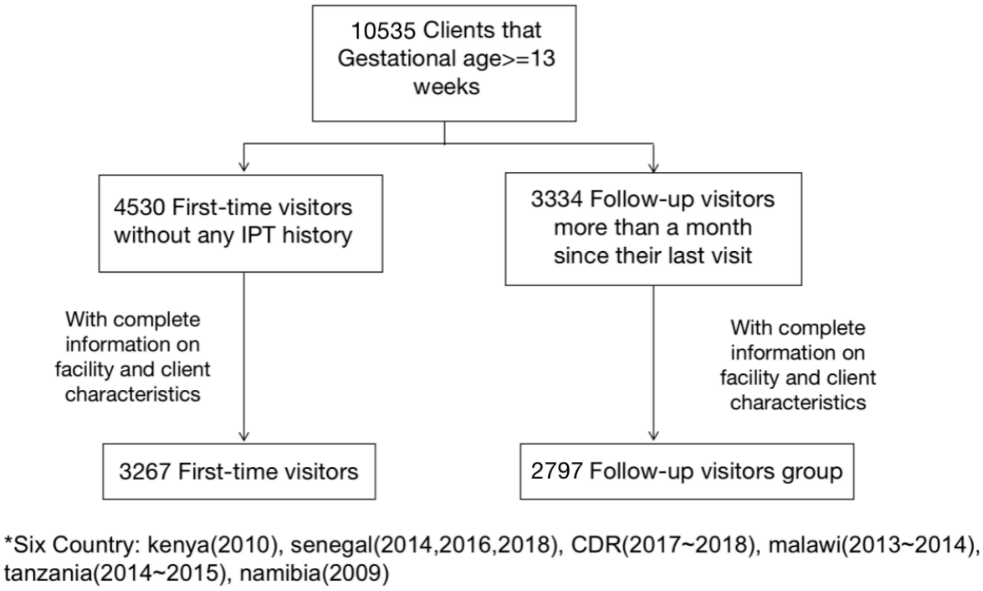

Figure S1. Flow chart of sampling

Abbreviation: ANC, antenatal care; IPTp, intermittent preventive treatment in pregnancy
